# A Novel *BTK* Gene Mutation in a Child With Atypical X-Linked Agammaglobulinemia and Recurrent Hemophagocytosis: A Case Report

**DOI:** 10.3389/fimmu.2019.01953

**Published:** 2019-08-20

**Authors:** Shu-Ping Han, Yung-Feng Lin, Hui-Ying Weng, Shih-Feng Tsai, Lin-Shien Fu

**Affiliations:** ^1^Department of Pediatrics, Taichung Veterans General Hospital, Taichung, Taiwan; ^2^Institute of Molecular and Genomic Medicine, National Health Research Institutes, Zhunan, Taiwan; ^3^Cancer Progression Research Center, National Yang-Ming University, Taipei, Taiwan; ^4^Institute of Genetics, National Yang-Ming University, Taipei, Taiwan; ^5^Department of Pediatrics, National Yang-Ming University, Taipei, Taiwan

**Keywords:** Bruton's tyrosine kinase, hemophagocytic lymphohistiocytosis, intravenous immunoglobulin, primary immunodeficiency disease, X-linked agammaglobulinemia

## Abstract

X-linked agammaglobulinemia (XLA), caused by a mutation in the Bruton's tyrosine kinase (*BTK*) gene, is rarely reported in patients with recurrent hemophagocytic lymphohistiocytosis (HLH). This mutation leads to significantly reduced numbers of circulatory B cells and serum immunoglobulins in patients. Therefore, they exhibit repetitive bacterial infections since infancy, and immunoglobulin (Ig) replacement therapy is the primary treatment. HLH is a life-threatening condition with manifestations of non-remitting fever, hepatosplenomegaly, cytopenias, coagulopathy, lipid disorder, and multiple organ failure. It is caused by the immune dysregulation between cytotoxic T cells, NK cells, and histiocytes. The treatment is based on HLH-2004 protocol including immunotherapy, chemotherapy, supportive therapy, and stem cell transplantation. However, as we know more about the classification and pathophysiology of HLH, the treatment is modified. T-cell-directed immunotherapy is effective in patients with primary HLH, and strong immunosuppression is contraindicated in patients with severe ongoing infections or some primary immunodeficiency diseases (PIDs). Here, we report the case of a 7-year-old boy who presented with ecthyma gangrenosum and several episodes of pyogenic infections during childhood. At the age of 5 years, he exhibited cyclic HLH every 2–3 months. The remission of HLH episodes finally achieved after he received monthly Ig replacement therapy (400 mg/kg) at the 4th HLH. However, transient elevation of IgM was incidentally discovered after 6 cycles of monthly Ig replacement therapy. IgM-secreting multiple myeloma, Waldenström's macroglobulinemia, and lymphoma were excluded. The IgM levels then declined and returned to the normal range within a year. The patient and his parents received whole-genome sequencing analysis. It revealed a novel hemizygous c.1632-1G>A mutation in the *BTK* gene and XLA was diagnosed. XLA exhibits a spectrum of clinical and immunological presentations in patients. The identification of the mutation in the *BTK* gene contribute to an accurate diagnosis. Ig replacement therapy is the primary treatment for HLH in patients with XLA.

## Introduction

### Background

X-linked agammaglobulinemia (XLA) is a primary immunodeficiency disease (PID) characterized by hypogammaglobulinemia with a small number of peripheral circulating mature B cells (<1%) in a male patient (1). It is caused by a mutation in the Bruton's tyrosine kinase (*BTK*) gene that leads to the failure of B cells to develop from the pro-B to pre-B stage. Patients present with frequent bacterial infections from the age of 6 months as their mothers' protective transplacental immunoglobulin (Ig) G depletes. From early childhood, they exhibit recurrent respiratory infections such as sinusitis, pneumonia, and otitis media; severe bacterial infections such as septicemia, osteomyelitis, and meningitis may also occur. They can fight most viral infections well because of the preserved number and function of T cells, but they are susceptible to hepatitis viruses and some enteroviruses. XLA is associated with some autoimmune diseases, malignancy, and growth hormone deficiency (2). It is safe and effective to treat patients with XLA with immunoglobulin (Ig) replacement therapy 400–600 mg/kg every 3–4 weeks to prevent severe infections (3). Hematopoietic stem cell transplantation in this patient group has not been fully studied and is not yet recommended, unless the patient presents with a malignant hematologic disease (4).

Hemophagocytic lymphohistiocytosis (HLH) is a life-threatening hyperinflammation caused by immune dysregulation between cytotoxic T cells, NK cells, and histiocytes, resulting in non-remitting fever, hepatosplenomegaly, cytopenias, coagulopathy, lipid disorder, and organ infiltration by activated macrophages performing phagocytosis (5, 6). Primary HLH is caused by highly activated T cells with defective cytotoxicity. Patients with primary HLH have genetic mutations related to: (1) granule-mediated cytotoxic T and NK cells that result in familial HLH (*PRF1, UNC13D, STXBP2, STX11*); (2) lysozyme function that results in Chédiak–Higashi syndrome (*LYST*), Griscelli syndrome type 2 (*RAB27A*), and Hermansky–Pudlak syndrome 2 (*AP3B1*); and (3) the inhibited cytotoxic responses of cytotoxic T and NK cells to B cells infected by Epstein–Barr virus (EBV) that result in X-linked lymphoproliferative syndrome (*SH2D1A, XIAP*). Though rarely occurring, other PIDs have been reported to cause HLH, including combined immunodeficiency, chronic granulomatous disease, autoinflammatory diseases, and antibody deficiencies (5–9). In addition, a viral infection may trigger a flare-up in a patient with primary HLH. More Taiwanese patients with HLH have a disease type relevant to EBV infection (10). Secondary HLH, also known as macrophage activation syndrome, is present in patients undergoing severe infections, malignancies, autoimmune, or autoinflammatory diseases (7–10). It is challenging to distinguish between primary and secondary HLH by the initial presentations of a patient. The treatment is based on HLH-2004 protocol, including chemoimmunotherapy (corticosteroids, etoposide, cyclosporin A, and/or intrathecal methotrexate), prophylactic antibiotics, and intravenous Ig (IVIg). Hematopoietic stem cell transplantation is indicated in selected patients with refractory and/or relapsed disease after appropriate chemoimmunotherapy (11–13). However, it may be inappropriate and harmful to treat with aggressive immunosuppression in the patients with severe ongoing infections or PIDs other than familial HLH and X-linked lymphoproliferative syndrome. Furthermore, more T-cell targeting therapies, such as anti-thymocyte globulin, etoposide, and alemtuzumab, can be effective in patients with primary HLH caused by the overactivated T cells (8, 9).

### Case Presentation

**Figure 1:**
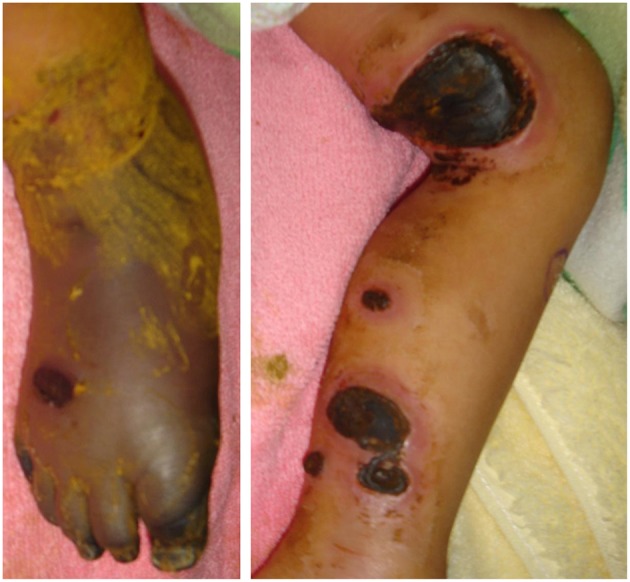
The ecthyma gangrenosum with poor peripheral perfusion over the patient's bilateral legs.

This 7-year-old boy suffered his first severe infection when he was aged 1 year, presenting with fever, vomiting, and diarrhea for 2 days, followed by the appearance of multiple enlarged erythematous rashes on the head, trunk, and limbs. The skin rash in his lower legs progressed, and some lesions became necrosis (Figure 1). The diagnosis of ecthyma gangrenosum was established on the basis of all available cultures yielding wild-type *Pseudomonas aeruginosa*. He also had septic shock; therefore, he received fluid resuscitation, meropenem administration, inotropic agents infusion, and endotracheal tube insertion with mechanical ventilation at intensive care unit. The necrotic wounds gradually improved and became scarred. Since then, he has had bacteremia, pyogenic pneumonia, sinusitis, and osteomyelitis several times. At the age of 5 years, he experienced 4 episodes of HLH over a period of 9 months that presented as persistent fever; hepatosplenomegaly; pancytopenia; disseminated intravascular coagulation; and increased levels of triglycerides, ferritin, and soluble interleukin-2 receptor. Moreover, he had chronic hepatitis B in the immune tolerance stage for years under lamivudine treatment. The patient's family medical history included the death of his mother's brother in infancy from an unknown disease and his mother being a hepatitis B virus carrier. Physical examination indicated the patient had failure to thrive, normal hair, intact skin except previous wound scars, a faint Bacillus Calmette–Guérin vaccine scar, invisible tonsils, and hepatosplenomegaly. His laboratory data were summarized in Table 1. The first HLH episode subsided soon after the infusion of IVIg (1 g/kg/day for 2 days). He did not undergo chemotherapy, as per the HLH protocol. He received oral prednisolone 2 mg/kg/day for 28 days followed by a gradual reduction in prednisolone dosage within 21 days. However, HLH recurred 1 month later after prednisolone was discontinued. The report from the bone marrow biopsy detailed non-caseating granulomatous inflammation with hemophagocytosis and the absence of a pathogen growing in the culture of the bone marrow. The second HLH subsided under IVIg therapy (1 g/kg/day for 2 days). He also received a 28-day course of oral prednisolone (2 mg/Kg/day) and then a 21-day course of prednisolone tapering. He was then kept on a low dose of prednisolone 0.25 mg/Kg every day for 3 weeks. The third HLH still recurred, and it also subsided quickly after IVIg therapy (1 g/kg/day for 2 days). He took prednisolone 2 mg/Kg/day for 14 days, with dose tapering within the next 28 days. Then he had the 4th episode of HLH around 1 month later. The first available data of Ig levels, checked 1 month after the infusion of IVIg (1 g/kg/day for 2 days) at the second episode of HLH, indicated the following: IgG: 639 mg/dL, IgA < 10.46 mg/dL, IgM < 1.09 mg/dL. The Ig data at the third episode of HLH before the commencement of IVIg infusion showed IgG: 236 mg/dL, IgA 18.1 mg/dL, and IgM 7.9 mg/dL, and the lymphocyte subsets exhibited no detectable B cells. The follow up data at the fourth episode of HLH were as follows: IgG: 259 mg/dL, IgA <10.46 mg/dL, and IgM <1.09 mg/dL with very few B cells (0.02%). The viral DNA PCR of herpes simplex virus-1, varicella zoster virus, EBV, cytomegalovirus, and parvovirus B19 were checked at the 4th episode of HLH showed negative results.

**Table 1:** The criteria and investigations of the index case for hemophagocytic lymphohistiocytosis and X-linked agammaglobulinemia.

**Hemophagocytic lymphohistiocytosis (HLH)**										**X-linked agammaglobulinemia (XLA)**	
**At least 5 of the following 8 findings:** **1. Non-remitting fever ≥38.5**^**°**^C **2. Splenomegaly** **3. Cytopenia: Hb ≤ 9 g/dL, Plt ≤ 100 × 1,000/μL, ANC ≤ 1,000/μL (at least 2)** **4. Hypofibrinogenemia (<150 mg/dL) or hypertriglyceridemia (≥265 mg/dL)**							**5. Hyperferritinemia (≥500 ng/mL)** **6. Increased level of soluble CD25 (sIL-2R)** **7. Evidence of hemophagocytosis in BM, LN, spleen, or liver** **8. Decreased or absent NK cell cytotoxicity**.			**Hypogammaglobulinemia and** **near absence of peripheral circulating B cells**	
	**Fever**	**Splenomegaly**	**Cytopenia**	**Fibrinogen** **(mg/dL)**	**TG** **(mg/dL)**	**Ferritin** **(ng/mL)**	**sIL-2R**^a^ **(pg/mL)**	**Hemophagocytosis**	**Other survey**	**Ig levels (mg/dL)**	**Lymphocyte subset**
1st HLH	+	+	+	95.2	344	1,214	>5,000	Not performed	EB VCA-IgG: 1.142^b^ EB VCA-IgM, EB-NA Ab, CMV-IgM, CMV-IgG: Neg.		
2nd HLH	+	+	+	80.9	270	791	>5,000	BM	BM culture: No pathogen growing.	IgA: < 10.46^c^ IgG: 639^c^ IgM: < 1.09^c^	
3rd HLH	+	+	+	131	213	690	4,263	Not performed		IgA: 18.1 IgG: 236 IgM: 7.9	T-cell: 98.52%, B-cell: ND, NK-cell: 1.48%
4th HLH	+	+	+	81.3	308	503	3,322	Not performed	Viral DNA PCR of HSV-1, VZV, EBV, CMV, parvovirus B19: Neg.	IgA: <10.46 IgG: 259 IgM: <1.09	T-cell: 99.98%, B-cell: 0.02%, NK-cell: N/A

+, present;

a Negative, < 880 pg/mL;

b Cuff-off-value, 1.1;

c *The data were checked 1 month after the infusion of immunoglobulin, 1 g/kg/day for 2 days, at the 2nd HLH, ANC, absolute neutrophil count*;

*BM, bone marrow; CMV, cytomegalovirus; EBV, Epstein–Barr virus; EB VCA-IgG, Epstein–Barr virus viral capsid antigen immunoglobin G; EB VCA-IgM, Epstein–Barr virus viral capsid antigen immunoglobin M; EB-NA Ab, Epstein–Barr virus nuclear antigen antibody; Hb, hemoglobin; HLH, Hemophagocytic lymphohistiocytosis; HSV, herpes simplex virus; Ig, immunoglobulin; LN, lymph node; N/A, Not available; ND, Non-detectable; Neg, Negative; PCR, polymerase chain reaction; Plt, platelet; TG, triglyceride; sIL-2R, soluble interleukin-2 receptor; VZV, varicella zoster virus*.

**Figure 2:**
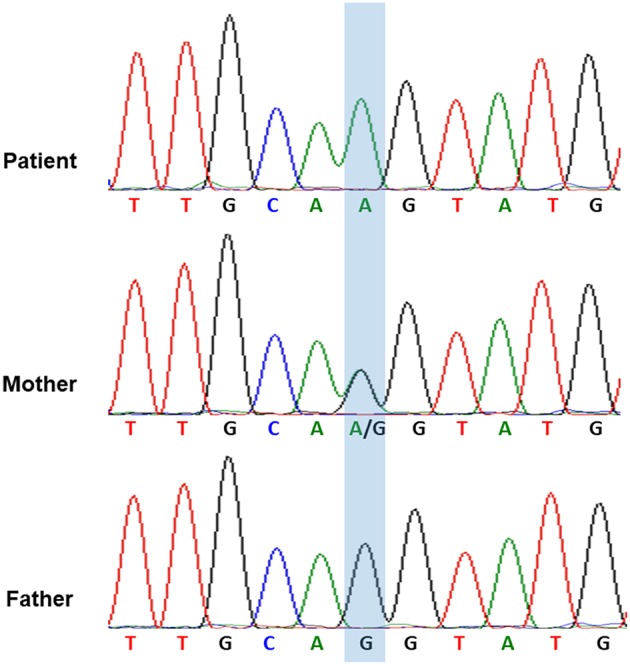
The genetic analysis of BTK gene indicated that the hemizygous c.1632-1G>A mutation of *BTK* gene in the patient and the heterozygous c.1632-1G>A mutation of *BTK* gene in his mother.

Considering the repeated bacterial infections experienced by the patient since infancy, as well as the recurrent episodes of HLH, global hypoglobulinemia, and the depletion of circulatory B cells, a PID entailing a B-cell defect, especially an X-linked defect, was highly suspected. The patient started regular Ig replacement therapy (400 mg/kg) every 4 weeks without receiving prednisolone after the 4th episode of HLH, which prevented further development of HLH attacks or the severe infections. After a 6-month course of monthly replacement of Ig (400 mg/kg), a significantly increased level of IgM (1,273 mg/dL) was discovered during a regular blood check-up. An IgM kappa monoclonal band was found in his serum immunofixation exam Table S1 in Supplementary Material. We performed whole genome sequencing analysis of the patient and both his parents. Genetic analysis revealed a novel hemizygous c.1632-1G>A mutation in the *BTK* gene of the patient (Figure 2). He was diagnosed to have XLA, and his mother is the carrier.

### Method

Whole-genome sequencing was performed using Illumina Novaseq 6000 System (Illumina Inc., San Diego, CA, USA). A novel hemizygous c.1632-1G>A mutation in the *BTK* gene was discovered. This mutation was confirmed by Sanger sequencing.

The study was approved by the Institutional Review Board I&II of Taichung Veterans General Hospital, Taiwan (No. CF17231A), and written informed consent for publication of this case report was obtained from the parents of the patient.

### Discussion

**Figure 3:**
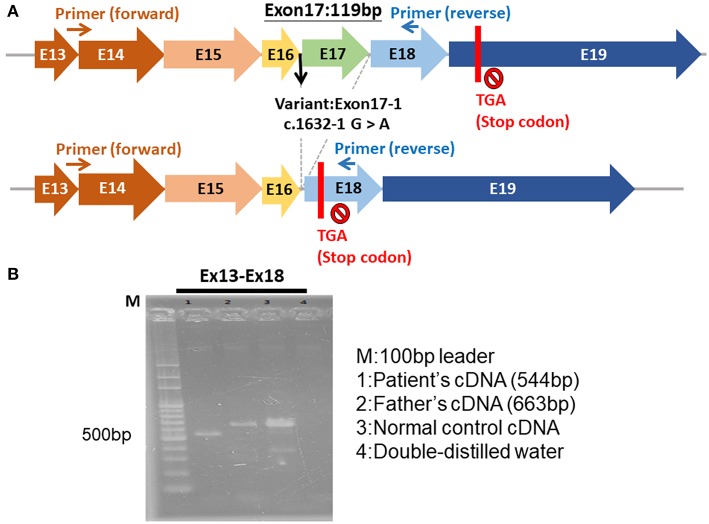
The hemizygous c.1632-1G>A mutation of *BTK* gene. **(A)** The exon 17 was skipped, leading to a frame-shift and premature termination. **(B)** The electrophoresis of the cDNA of *BTK* gene of the patient (544 bp), patient's father (663 bp), normal control, and double-distilled water.

The genetic study of our patient revealed a novel hemizygous c.1632-1G>A mutation in the *BTK* gene on the X chromosome. This mutation affects the mRNA splice of the *BTK* gene, causing exon 17 to be skipped and leading to a frame-shift and premature termination codons (Figures 3A,B). Few reports have addressed HLH in patients with XLA (14, 15). Two male siblings with XLA and the *BTK* mutation were reported to exhibit HLH after adenovirus infection (14). One of the siblings died from the uncontrolled dissemination of adenovirus infections after receiving chemotherapy for HLH. The other sibling developed HLH later and survived because of the administration of Ig therapy instead of chemotherapy; he remained healthy under regular Ig replacement therapy (14). Another case report documented a 27-year-old man with undetectable circulatory B cells and selective IgM deficiency who exhibited HLH, which resolved after treatment with Ig replacement therapy (400 mg/kg), dexamethasone, and cyclosporine (15). The mechanism through which *BTK* mutation induces HLH remains unclear, but some studies have demonstrated that *BTK* participates in the activation of innate immunity through its involvement in the Toll-like receptors signaling pathway (16, 17).

The disease severity of XLA is influenced by the specific mutation in the *BTK* gene. Some *BTK* mutations can preserve some *BTK* enzyme activity, which is related to detectable circulating B cells, higher immunoglobulin levels, less severe clinical manifestations, and delayed diagnosis (18, 19). Thus, the transient elevated IgM in our patient can be partially explained. Other studies have reported the patients with atypical XLA with normal or near-normal levels of 1 or more certain immunoglobulin isotype(s) (19–21). These atypical XLA phenotypes were indistinguishable from other PIDs. Thus, the use of genetic analysis facilitates an accurate diagnosis.

The initial immunoglobulin data of our patient were obtained 1 month after the IVIg infusion (1 g/kg/day for 2 days) was administered to treat the second episode of HLH. He had undetectable plasma IgM and IgA levels, but the IgG level was within the normal range at that time. However, the IgG levels checked at the subsequent episodes of HLH showed a significant decrease before the commencement of IVIg treatment (1 g/kg/day for 2 days). The mutation in our patient was classified as a severe mutation because it occurred at the invariant sites of the splicing consensus sequence—the first and last 2 base pairs of the intron, which was consistent with the considerably decreased levels of all immunoglobulins in our patient. However, the significant increase in the IgM levels after a 6-month course of successive monthly Ig replacement (400 mg/Kg) suggested some mature B-cells were preserved in the patient (22). Moreover, it reminded us of the possibility of malignant change in a patient with immunodeficiency. IgM-secreting multiple myeloma and Waldenström's macroglobulinemia, both of which are very rare in adult patients and not seen in pediatric patients, were excluded because the plasma cells were <2% in the bone marrow biopsy survey. No evidence of lymphoma was discernible in the gallium scan. In the currently available publications, no IgM-secreting lymphoma, multiple myeloma, or Waldenström's macroglobulinemia has been reported in patients with XLA. The patient continued receiving monthly Ig infusions (400 mg/Kg) with the same dose and brand. IgM levels declined in the serial follow-ups and returned to the normal range 1 year later.

## Conclusion

There is a broader range of clinical and immunological manifestations in XLA patients. Neither normal nor significantly increased immunoglobulin levels can exclude XLA. The analysis of the *BTK* gene helps to facilitate the accurate diagnosis. Additionally, HLH can be one of the severe complications of XLA. Physicians should be alert and consider Ig replacement therapy (400–600 mg/Kg every 3–4 weeks) to be the primary therapy for this condition.

## Data Availability

The datasets for this manuscript are not publicly available because the containing information affects the privacy of research participants. Requests to access the datasets should be directed to the corresponding author L-SF, linshienfu@yahoo.com.tw.

## Ethics Statement

This study was carried out in accordance with the recommendations of Institutional Review Board I&II of Taichung Veterans General Hospital, Taiwan, with written informed consent from all subjects. All subjects gave written informed consent in accordance with the Declaration of Helsinki. The protocol was approved by the Institutional Review Board of the Taichung Veteran General Hospital, Taiwan (No. CF17231A).

## Author Contributions

S-PH provided medical care to the patient, conceptualized the case report, collected data, and drafted the initial manuscript. S-FT, Y-FL, and H-YW participated in the genetic analysis, molecular biological experiment, and revised the manuscript in the genetic part. L-SF reviewed and revised the manuscript. All authors approved the final manuscript as submitted and agree to be accountable for all aspects of the work.

### Conflict of Interest Statement

The authors declare that the research was conducted in the absence of any commercial or financial relationships that could be construed as a potential conflict of interest.
