# Supplementary material for: A Novel BTK Gene Mutation in a Child With Atypical X-Linked Agammaglobulinemia and Recurrent Hemophagocytosis: A Case Report
Source: Front Immunol. 2019 Aug 20;10:1953. doi: 10.3389/fimmu.2019.01953 (PMC6711359; doi:10.3389/fimmu.2019.01953)
Supplement: Supplementary file 1 [file Data_Sheet_1.PDF]

Supplementary Material

1 Supplementary Table 1. The survey for EBV infection.

	Nov. 2015<br>1st HLH	Aug. 2016<br>4th HLH	Apr. 2017<br>Hyper-IgM stage<br>(IgM: 566mg/dL)	Jul. 2018<br>Serum IgM within normal range
EB VCA IgM	Negative	Not performed	Negative	Negative
EB VCA IgG	Positive (weak)	Not performed	Positive (weak)	Positive
EBNA Ab	Negative	Not performed	Borderline	Positive
EBV PCR	Not performed	Negative	Not performed	Not performed
Before or after IVIg infusion during the hospitalization	Before	Before	Before	After
